# Inflammation in Cachexia

**DOI:** 10.1155/2015/536954

**Published:** 2015-10-07

**Authors:** M. Seelaender, A. Laviano, S. Busquets, G. P. Püschel, T. Margaria, M. L. Batista

**Affiliations:** ^1^Cancer Metabolism Research Group, Institute of Biomedical Sciences, University of São Paulo, Avenida Professor Lineu Prestes 1524, 05508-900 São Paulo, SP, Brazil; ^2^Department of Clinical Medicine, Sapienza University, Viale dell'Università 37, 00185 Rome, Italy; ^3^Cancer Research Group, Department of Biochemistry and Molecular Biology, Biology Faculty, University of Barcelona, Avenida Diagonal 643, 08028 Barcelona, Spain; ^4^Department of Nutritional Biochemistry, Institute of Nutritional Science, University of Potsdam, Arthur-Scheunert-Allee 114, 11614558 Nuthetal, Germany; ^5^Lero, The Irish Software Research Center, University of Limerick, Limerick, Ireland; ^6^Laboratory of Adipose Tissue Biology, Integrated Group of Biotechnology, University of Mogi das Cruzes, Avenida Dr. Cândido Xavier de Almeida Souza 200, Centro Cívico, 08780-911 Mogi das Cruzes, SP, Brazil

Cachexia is a complex wasting syndrome associated with a marked detrimental effect upon life quality and survival in patients with cancer, chronic obstructive pulmonary disease (COPD), chronic heart failure, AIDS, and chronic kidney disease, among other conditions. Its prevalence is of around 5 to 15% in cardiac patients at end stage, rising up to 30%, in COPD and chronic kidney disease patients, and to 80%, in patients with advanced cancer. Cachexia symptoms include pronounced weight loss, due to both lean and fat mass wasting: anorexia, malabsorption, nausea, asthenia, neuroendocrine changes, immune system function impairment, and disruption of energy metabolism. Despite its unquestionable relevance to the poorer outcome of treatment in disease and its high prevalence among patients, the syndrome is still underdiagnosed and seldom treated. Part of the difficulty in treating cachexia relies on the fact that, in the clinical setting, the syndrome is recognised solely in its most advanced stages, when therapy available to the present day is not able to fully reverse its symptoms. Therefore, scientists and clinicians should focus on identifying early changes, as to intervene in a precocious manner.

The aetiology of cachexia has not been fully unveiled, yet it appears that chronic systemic inflammation is present in the vast majority of patients. The aim of the present special issue is to address the importance of systemic inflammation in cachexia, in regard to its consequences and to its possible role in providing early markers for the diagnosis of the syndrome.

Cancer cachexia-related neuroinflammation is discussed by A. Molfino et al., as the authors propose a conceptual framework in which the hypothalamus transduces the peripheral challenge represented by the presence of the tumour into catabolic signals, as a result of central inflammation. A contribution by N. Inácio Pinto et al. examines the role of inflammatory signalling factors involved in the communication among the peripheral tissue, tumour microenvironment, and the central nervous system. Another view of such interactions is provided by J. M. Argiles et al., who bring similar emphasis on the conversation among different body compartments and organs in cancer cachexia. The authors comment on the significance of tissues other than the skeletal muscle in the mechanisms underlying the syndrome, proposing that the latter suffers wasting as a consequence of systemic inflammatory changes. Adding information on the role of inflammatory factors on muscle wasting, D. Costamagna et al. discuss the molecular mechanisms involved in muscle homeostasis disruption and mass loss.

The quest for markers of the initiation of cachexia is also debated: M. Ebadi and V. C. Mazurak propose the adoption of adipose tissue-derived factors as indicators of early inflammatory alterations that induce fat mass wasting in the syndrome. R. Camargo et al. review the potential of microRNAs in the regulation of cancer-cachexia systemic inflammation and put forward the possibility that these molecules may serve as diagnostic tools. Finally, the article by D. Watt et al. presents the convenience and adequacy of employing prognostic scores that include systemic inflammation assessment as a valuable means for cachexia diagnosis.

Taken together, the issue provides insights on the importance of detecting early signs of inflammatory changes in patients and examines the mechanisms that act in concert, inducing cachexia symptoms.

*M. Seelaender* *M. Seelaender* *A. Laviano* *A. Laviano* *S. Busquets* *S. Busquets* *G. P. Püschel* *G. P. Püschel* *T. Margaria* *T. Margaria* *M. L. Batista Jr.* *M. L. Batista Jr.*

